# Supplementary figures and images for: IL-4/IL-13 polarization of macrophages enhances Ebola virus glycoprotein-dependent infection
Source: PLoS Negl Trop Dis. 2019 Dec 11;13(12):e0007819. doi: 10.1371/journal.pntd.0007819 (PMC6905523; doi:10.1371/journal.pntd.0007819)

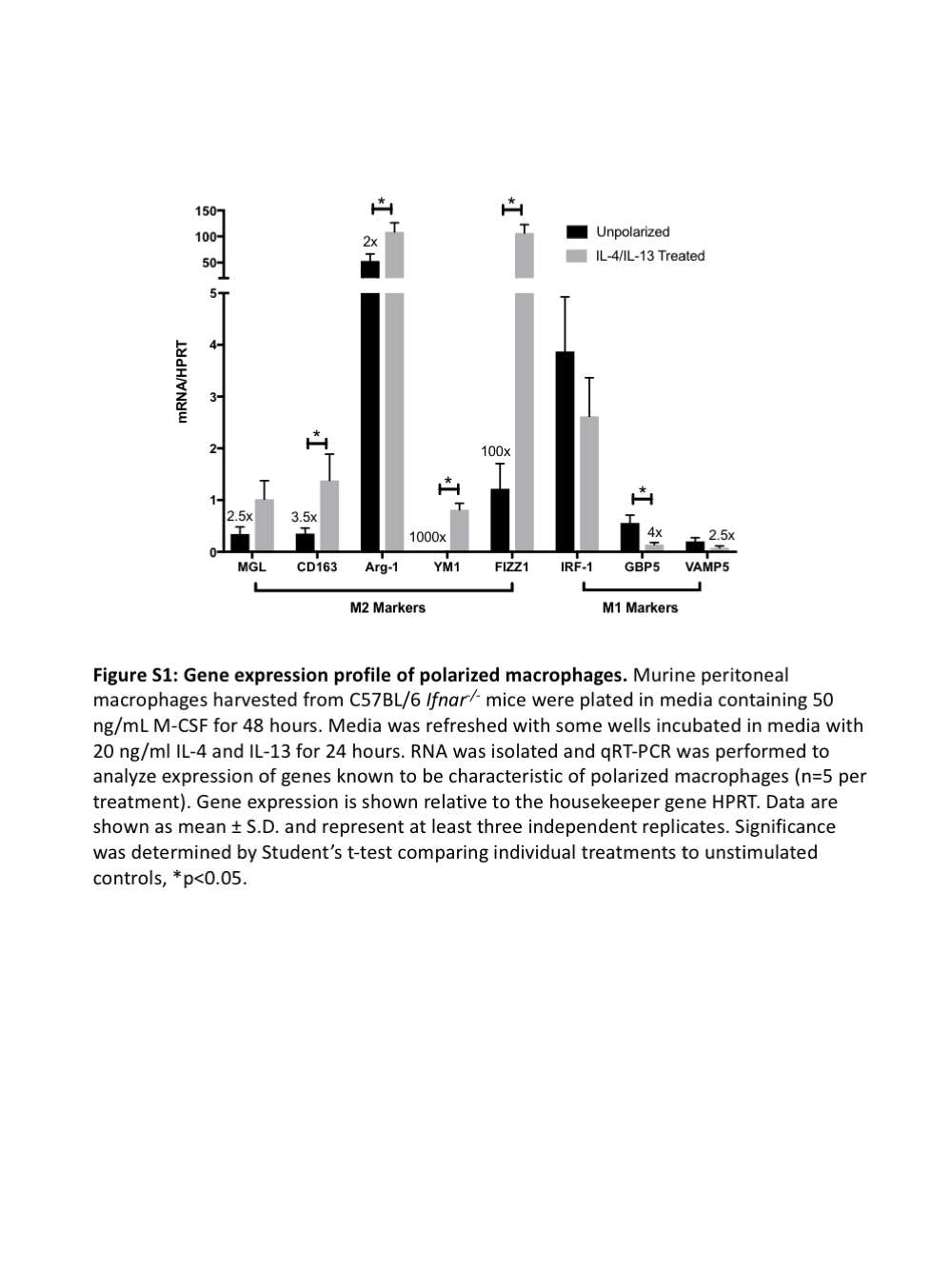

Supplement: S1 Fig — Murine peritoneal macrophages harvested from C57BL/6 Ifnar-/- mice were plated in media containing 50 ng/mL M-CSF for 48 hours. Media was refreshed with some wells incubated in media with 20 ng/ml IL-4 and IL-13 for 24 hours. RNA was isolated and qRT-PCR was performed to analyze expression of genes known to be characteristic of polarized macrophages (n = 5 per treatment). Gene expression is shown relative to the housekeeper gene HPRT. Data are shown as mean ± S.D. and represent at least three independent replicates. Significance was determined by Student’s t-test comparing individual treatments to unstimulated controls, *p<0.05. (TIF) [file pntd.0007819.s001.tif]

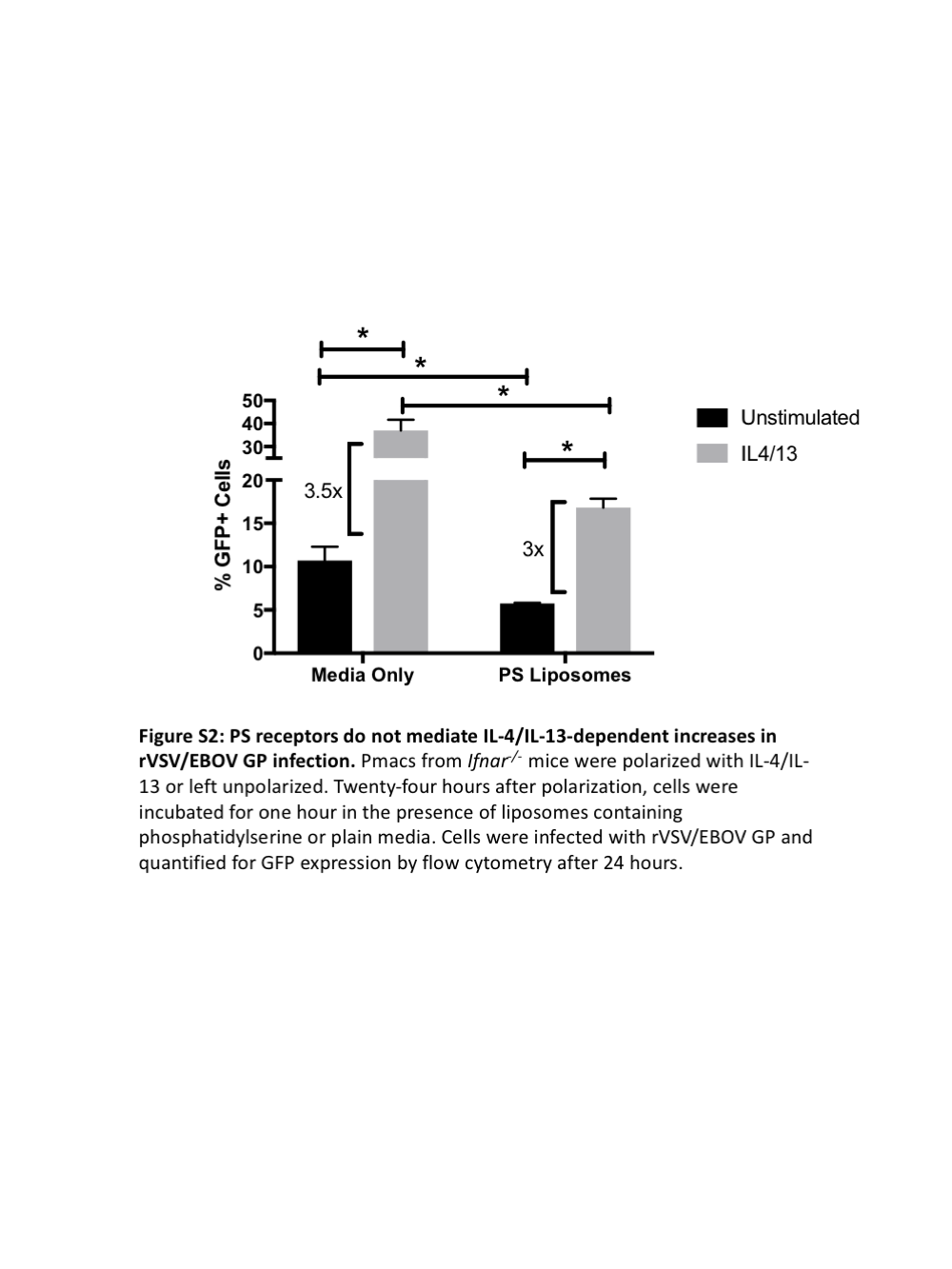

Supplement: S2 Fig — Pmacs from Ifnar-/- mice were polarized with IL-4/IL-13 or left unpolarized. Twenty-four hours after polarization, cells were incubated for one hour in the presence of liposomes containing phosphatidylserine or plain media. Cells were infected with rVSV/EBOV GP and quantified for GFP expression by flow cytometry after 24 hours. Data are shown relative to levels of infection in pmacs not stimulated with IL-4/-13. Statistics were performed with Student’s t-test, * indicates p value < 0.05. (TIF) [file pntd.0007819.s002.tif]

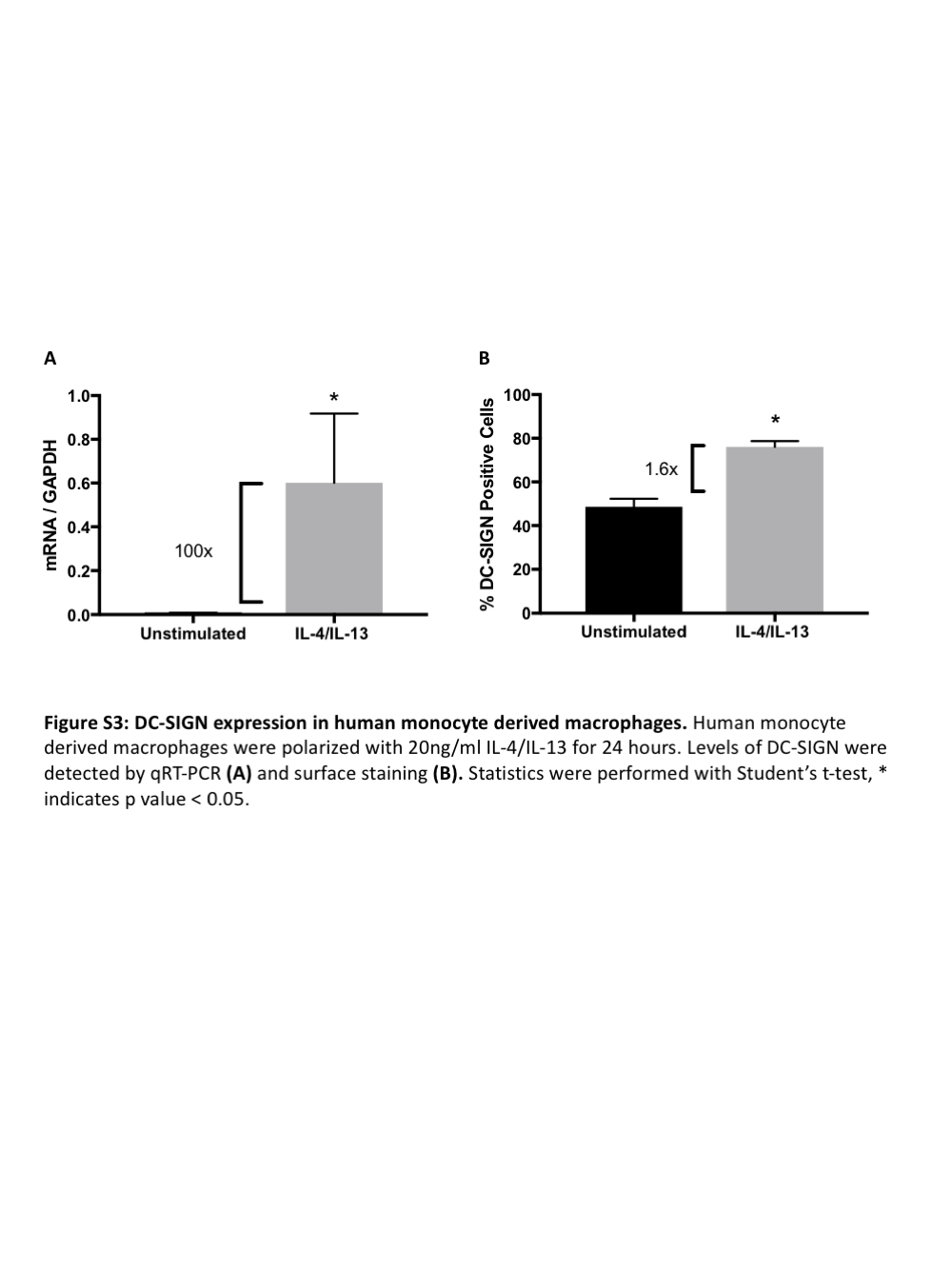

Supplement: S3 Fig — Human monocyte derived macrophages were polarized with 20 ng/ml IL-4/IL-13 for 24 hours. Levels of DC-SIGN were detected by qRT-PCR (A) and surface staining (B). Statistics were performed with Student’s t-test, * indicates p value < 0.05 (TIF) [file pntd.0007819.s003.tif]

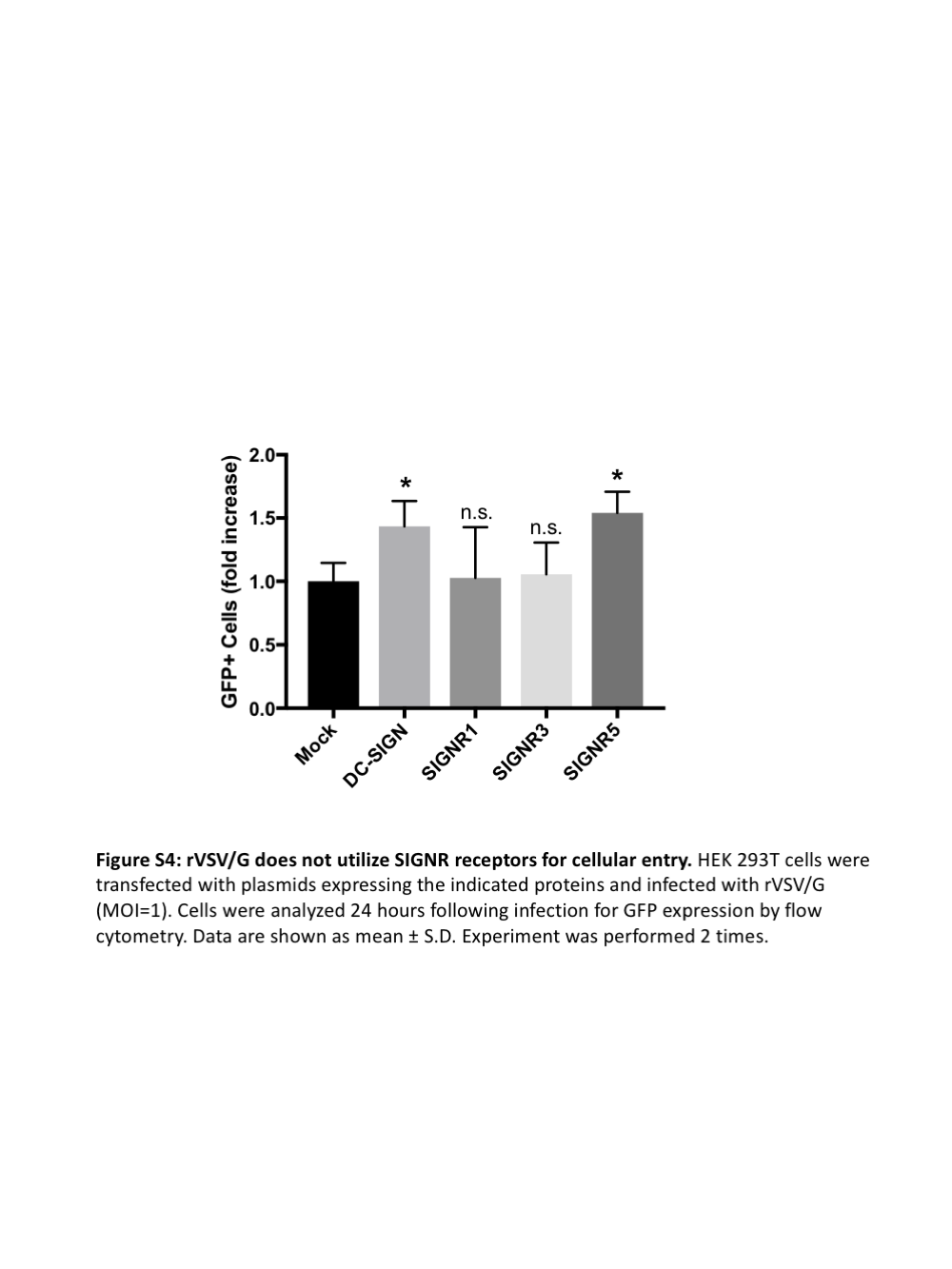

Supplement: S4 Fig — HEK 293T cells were transfected with plasmids expressing the indicated proteins and infected with rVSV/G (MOI = 1). Cells were analyzed 24 hours following infection for GFP expression by flow cytometry. Data are shown as mean ± S.D. Experiment was performed 2 times. Statistics were performed with Student’s t-test, * indicates p value < 0.05 (TIF) [file pntd.0007819.s004.tif]

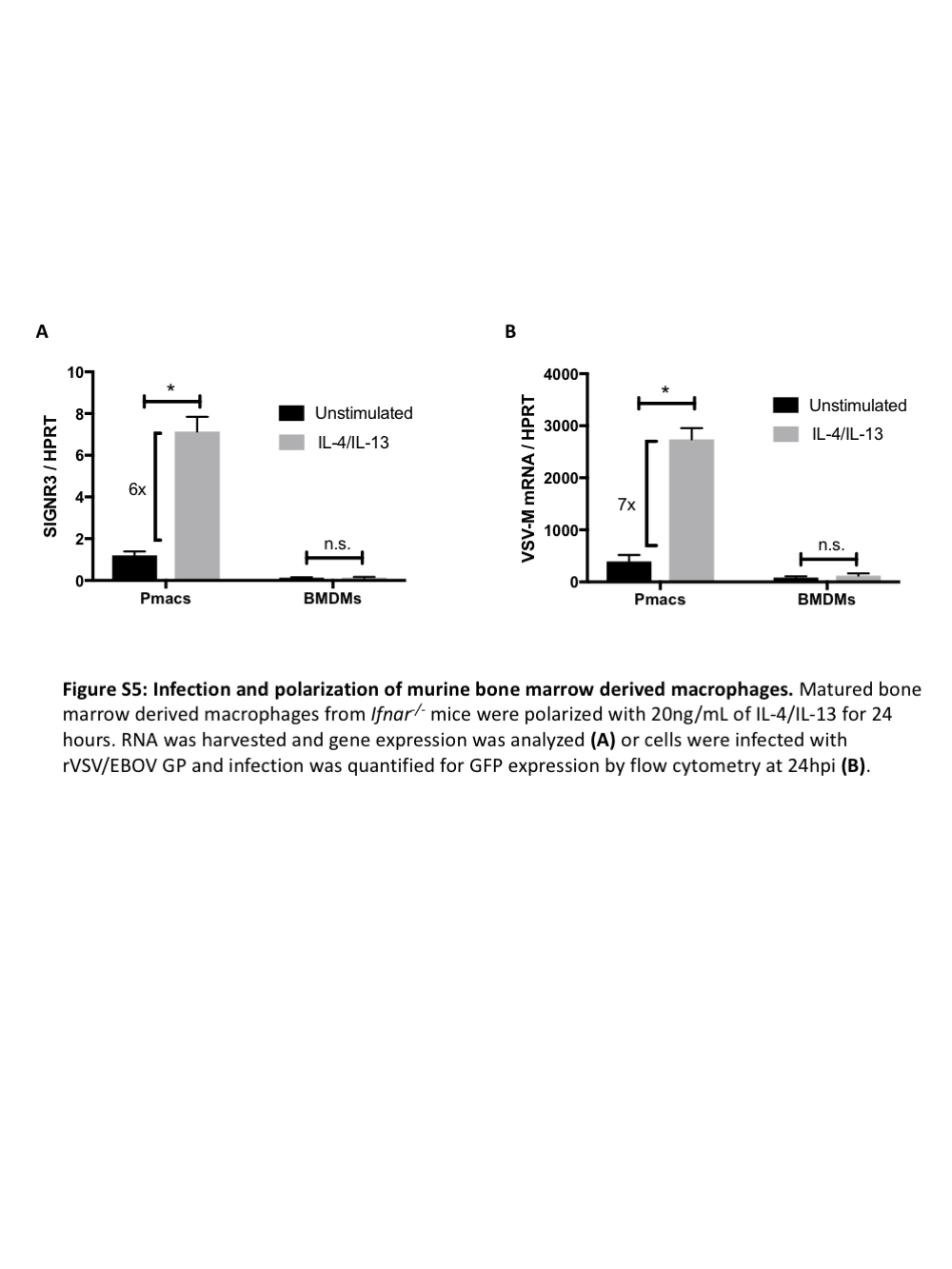

Supplement: S5 Fig — Matured bone marrow derived macrophages from Ifnar-/- mice were polarized with 20 ng/mL of IL-4/IL-13 for 24 hours. RNA was harvested and gene expression was analyzed (A) or cells were infected with rVSV/EBOV GP and infection was quantified for GFP expression by flow cytometry at 24hpi (B). Experiment was performed 2 times. Statistics were performed with Student’s t-test, * indicates p value < 0.05 (TIF) [file pntd.0007819.s005.tif]

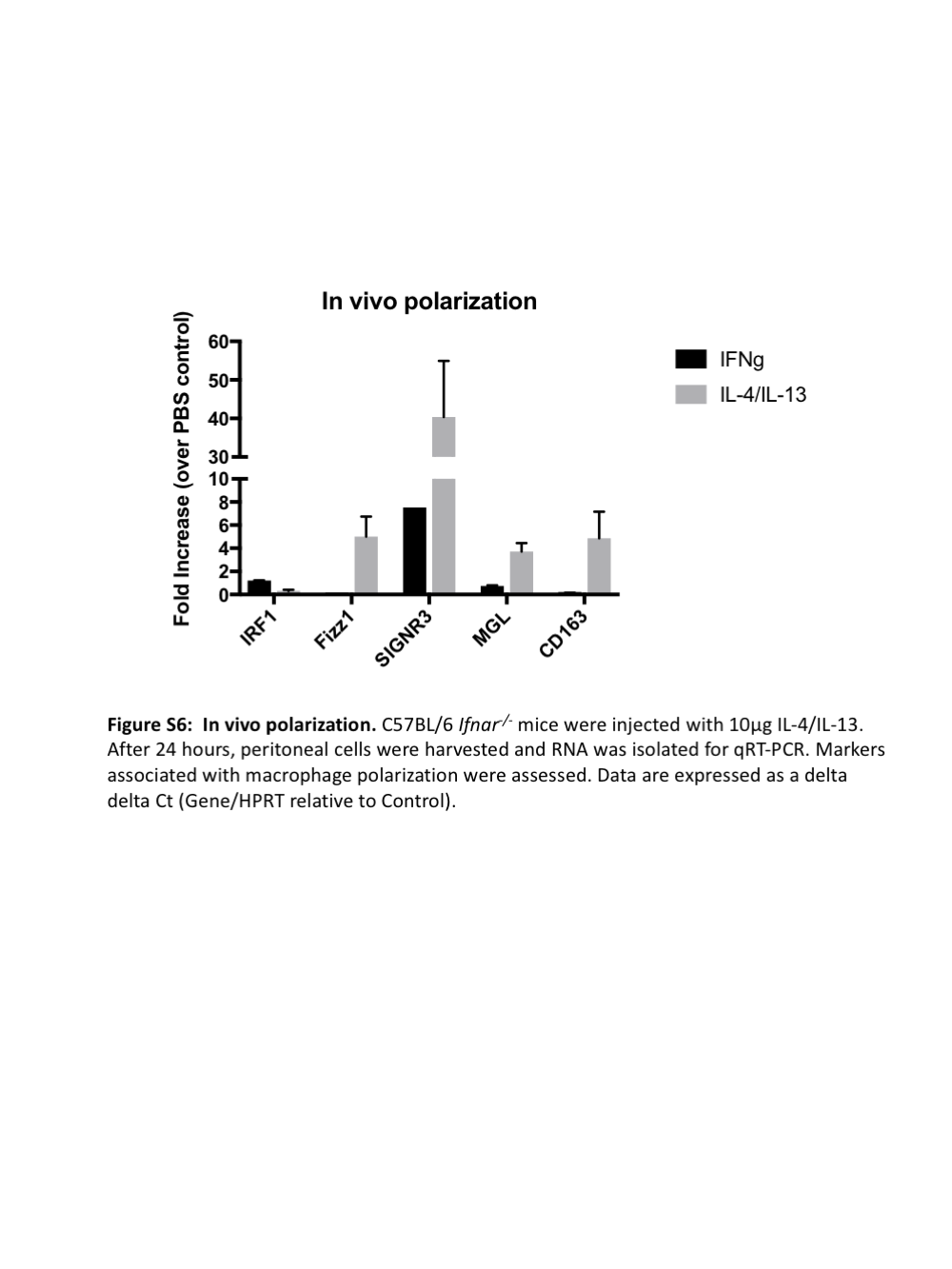

Supplement: S6 Fig — C57BL/6 Ifnar-/- mice were injected with 10 μg IL-4/IL-13. After 24 hours, peritoneal cells were harvested and RNA was isolated for qRT-PCR. Markers associated with macrophage polarization were assessed. Data are expressed as a delta delta Ct (Gene/HPRT relative to Control) (TIF) [file pntd.0007819.s006.tif]
