# Supplementary material for: IL-4/IL-13 polarization of macrophages enhances Ebola virus glycoprotein-dependent infection
Source: PLoS Negl Trop Dis. 2019 Dec 11;13(12):e0007819. doi: 10.1371/journal.pntd.0007819 (PMC6905523; doi:10.1371/journal.pntd.0007819)
Supplement: Attachment — Submitted filename: Author response.docx [file pntd.0007819.s007.docx]

**Response to Reviewers**

We would like to thank the reviewers for their thoughtful consideration of the manuscript and their valuable feedback. We feel this manuscript is strengthened by your comments. To facilitate your review of these comments we have included responses in red font below. Furthermore any text or figure changes made are indicated in addition to being highlighted in the text.

**Author Responses to Specific Reviewer Questions**

Reviewer #1: All OK; one instance where additional statistics are needed is noted in Specific Comments Thank you, this is now addressed (see below).

Reviewer #2: Lines 216-220: The authors state that “cultures were incubated for 24 h in the plating media without MCSF with: 20ng/mL IL-4 + 20ng/mL IL-13 (M2a), 150μg/mL immune complexes (10:1 ratio of anti-ova antibody/ova) and 10ng/mL LPS (M2b), 100nM Dexamethasone (M2c), or 20ng/mL interferon gamma (M1) for 24 hours”. Oddly, the M1, M2b, or M2c data is not presented in the manuscript. A direct infectivity comparison among these macrophage subsets would have made their findings more substantial. This is especially important considering the literature suggests EBOV elicits an LPS-like response via TLR4 activation (M2b-like response), and this mechanism is thought to be GP-dependent. IL-10 is also secreted during EBOV infection, which could facilitate M2c differentiation. Thank you for catching this editing error! Indeed, during these studies, we did compare multiple polarization states and, in our limited sampling of gene expression changes due to the polarization conditions, we found that our polarization conditions resulted in expected gene expression. However, because we only sampled only a few genes for the M1, M2b and M2c conditions, we chose not include those data in the submitted manuscript, but focused on the M2a phenotype. We have now removed these lines from the manuscript. The revised section begins on line 216. However, if the reviewer feels strongly about showing the data on gene expression following M1, M2b and M2c, these data can be included in supplemental information since they are readily available.

Per the reviewer’s comments about TLR4 interactions with EBOV GP, we have not explored the effect of rVSV/EBOV GP infection on polarization. In these kinds of experiments, we worried that virus-induced cytotoxicity might confound our results. However, the point that the reviewer makes is interesting and insightful. Indeed virus bearing EBOV GP may “push” macrophages towards a M2b phenotype as the infection progresses. Such studies should be performed in the future. This idea is now discussed in the paragraph beginning on line 583.

The peritoneal macrophages used in these studies were selected by adherence rather than FACS or magnetic sorting. This inferior method resulted in 85% purity. Thus, other cells could account for some of the observed effects even though they represent a smaller proportion of total cells. This point is well taken. We, and others, have phenotyped the peritoneal cavity and found that macrophages are a predominant cell type in the naïve peritoneal. Our studies also show that macrophages are the overwhelmingly predominant cell type infected by EBOV. While B cells also compose a similarly large component of the cavity, these cells were not found to be susceptible to infection. Interestingly, we do see a low, but reproducible, level of peritoneal eosinophils that appear to be infected. We plan to further study this observation in the future. Our data on the peritoneum composition and infection status of those cells are part of a separate manuscript that we currently have under review and therefore do not wish to include these data in this manuscript.

While it is certainly possible that cytokine production by B cells or other hematopoietic populations within the peritoneum may alter murine macrophage gene expression and thereby virus infection, our similar findings with IL-4/IL-13 treated human MDM that have been extensively washed and in vitro matured for 6 days makes this unlikely (Figure 1b).

--------------------

Reviewer #1: Yes, yes, yes

Reviewer #2: The results and figures were clearly presented and of sufficient quality. However, small groups (n=3) for some of the in vivo experiments may have skewed results. For example, Figure 7C and Figure 7D show ifnar/timd4-null mice given the same dose (1000 i.u.) of rVSV/EBOV GP exhibited 50% and 100% survival, respectively. Thank you for pointing this out. Your comment prompted additional studies. We have repeated these experiments (7D and 7E) to increase the n. These results remain statistically significant but more closely resemble the results in figure 7C.

Figure 7F states that a “representative” experiment was performed two independent times. Did this data match the other experiment exactly? The experiment has been performed again and the two experiments are compiled.

--------------------

Reviewer #1: yes, yes, yes, yes

Reviewer #2: The use of a surrogate virus (recombinant rVSV/EBOV GP) instead of wt EBOV is not ideal but appropriate for these studies considering the advantages of this model (i.e. safety, convenience, record for filovirus entry studies). However, the authors should discuss some of the limitations of this model and how the use of IFNAR deficient cells could skew their results. Thank you for pointing this out, we have added two new paragraphs to the discussion starting at line 498.

No reference is provided showing M2a-specific polarization during helminth infection to justify the focus on this subset (other than IL-4 secretion during EBOV infection). Other cytokines, such as IL-10, are also secreted during EBOV infection, which could polarize macrophages toward the M2c subset. Moreover, EBOV patients co-infected with malaria are 20% more likely to survive, despite this parasite being associated with an M2-skewed phenotype. Very interesting point! We actually have a manuscript under review looking at malaria and Ebola co-infections. This is an area of active interest for our lab. Additionally, we have added relevant references to line 133.

No explanation is provided for the lack of productive rVSV/EBOV GP infection in murine BMDM. Thank you for catching this. We believe this is due to lack of expression of TIM-4 on BMDMs, whereas it is highly expressed on Pmacs. This has been added to the manuscript on lines 440-441.

--------------------

Reviewer #1: Minor Revision as per Minor ('specific') comments

Reviewer #2: Minor revision. Please see above for specific comments.

--------------------

Reviewer #1:

Specific Comments
1.      It is suggested to include Supp. Fig 2 (effect IL4/13 treatment on VSV-EBOV GP infection of pMacs from WT mice) as a panel in Fig. 1 Agreed. This has now been moved to Figure 1.

2.      Line 359: suggested rewrite: “…enhances EBOV GP-mediated binding and entry” We agree that this sentence was poorly worded and have revised it. This is found beginning on line 357

3.      Line 371: suggest rewriting: Given the (stronger) enhancement of binding, one can’t say that there is also enhanced fusion per se. The increased entry signal could be because more VLPs are bound. This point is well taken. We have amended the statement to reflect this. This revision is found on line 368

4.      Fig. 2C (and other key Figs): Add the fold-increase notation (as in Fig 2A,B). We agree this makes data easier to interpret and have added this notation to every graph that is not reported as a fold change.

5.      Line 388-389: RE: “…suggests…PS receptors in pMacs are important for constitutive virus entry”: either delete or explain better. The statement re: constitutive virus entry does not seem to be supported by the Fig. (Supp. Fig. 3); although it is supported by the literature and subsequent Figures in this manuscript. Thank you for making this observation. We have changed the way we display these data to show that PS treatment reduced the magnitude of infection in both untreated and IL-4/IL-13 treated cells. However, the increase due to IL-4/IL-13 treatment is conserved in the presence of PS, suggesting that the IL-4/-13 dependent mechanism is PS independent, even though PS receptors are still important.

6.      Supp. Fig. 5 and Lines 409-410: provide statistics. (Are the ~1.5X upticks in GFP+ cells upon transfection of DC-SIGN or SIGNR5 statistically significant?) Thank you for pointing out this oversight on our part. Yes, the two groups achieved statistical significance although the magnitude of change is significantly reduced compared to the effect observed in rVSV/EBOV GP. We have now added this to the manuscript on lines 405-407.

7.      Line 452: Rephrase: The effect on VLP entry is small relative to effect on VLP binding. This has been added to the text at line 450.

Reviewer #2: Rogers et al. describe the cytokine milieu that promotes EBOV entry. IL-4 and IL-13 polarizes macrophages to an M2a phenotype, which enhances uptake of pseudotyped rVSV-EBOV-GP into murine peritoneal and human monocyte-derived macrophages. The proposed mechanism for this increased infectivity is increased expression of DC-SIGN (or DC-SIGN-like) molecules, which facilitate GP-mediated attachment and entry. Importantly, this work describes murine orthologs that may represent human DC-SIGN equivalents, a controversial topic in the immunology field that needs further clarification. In vivo co-administration of IL-4 and IL-13 increased mortality and enhanced pathogenesis in mice, illustrating the importance of M2a-skewing in the context of EBOV infection. The manuscript is well-written and the experimental design is sound. Moreover, the hypothesis is clearly stated and testable.
